# Comparative Lipidomic Analysis Reveals Heat Stress Responses of Two Soybean Genotypes Differing in Temperature Sensitivity

**DOI:** 10.3390/plants9040457

**Published:** 2020-04-04

**Authors:** Sruthi Narayanan, Zolian S. Zoong-Lwe, Nitant Gandhi, Ruth Welti, Benjamin Fallen, James R. Smith, Sachin Rustgi

**Affiliations:** 1 Department of Plant and Environmental Sciences, Clemson University, Clemson, SC 29634, USA; zzoongl@clemson.edu (Z.S.Z.-L.); bfallen@clemson.edu (B.F.); 2 Pee Dee Research and Education Center, Clemson University, Florence, SC 29506, USA; nitantg@clemson.edu; 3 NCJ Diagnostics & DNA Technologies, Monmouth Junction, NJ 08852, USA; 4 Kansas Lipidomics Research Center, Division of Biology, Kansas State University, Manhattan, KS 66506, USA; welti@ksu.edu; 5 USDA, Agricultural Research Service, Crop Genetics Research Unit, Stoneville, MS 38776, USA; rusty.smith@usda.gov

**Keywords:** soybean, heat stress, lipidomics, lipid metabolic changes, lipid unsaturation, fatty acid desaturase

## Abstract

Heat-induced changes in lipidome and their influence on stress adaptation are not well-defined in plants. We investigated if lipid metabolic changes contribute to differences in heat stress responses in a heat-tolerant soybean genotype DS25-1 and a heat-susceptible soybean genotype DT97-4290. Both genotypes were grown at optimal temperatures (OT; 30/20 °C) for 15 days. Subsequently, half of the plants were exposed to heat stress (38/28 °C) for 11 days, and the rest were kept at OT. Leaf samples were collected for lipid and RNA extractions on the 9^th^ and 11^th^ days of stress, respectively. We observed a decline in the lipid unsaturation level due to a decrease in the polyunsaturated linolenic acid (18:3) content in DS25-1. When examined under OT conditions, DS25-1 and DT97-4290 showed no significant differences in the expression pattern of the *Fatty Acid Desaturase* (*FAD*) *2-1A*, *FAD2-2B*, *FAD2-2C*, *FAD3A* genes. Under heat stress conditions, substantial reductions in the expression levels of the *FAD3A* and *FAD3B* genes, which convert 18:2 lipids to 18:3, were observed in DS25-1. Our results suggest that decrease in levels of lipids containing 18:3 acyl chains under heat stress in DS25-1 is a likely consequence of reduced *FAD3A* and *FAD3B* expression, and the decrease in 18:3 contributes to DS25-1′s maintenance of membrane functionality and heat tolerance.

## 1. Introduction

Soybean is one of the most important oilseed crops and an affordable source of plant proteins worldwide [1]. Currently, the U.S. is the largest soybean producer in the world, accounting for 34% of total production, followed by Brazil (32%) and Argentina (15%) [1]. High temperature was identified as a critical environmental factor limiting soybean yield worldwide [2]. An increase of 1 °C during the growing season may result in a 17% decrease in yield [3]. Heat stress causes soybean yield suppression of up to 6% daily under rainfed conditions when the growing season temperature is above 30 °C [4]. Soybean, which is predominantly a rainfed crop, often experiences high temperatures during its growing season. Therefore, it is anticipated that with climate change, heat stress will become a serious threat to soybean production [4]. Thus, understanding the mechanisms of heat tolerance is critical to developing climate-resilient soybean varieties.

Lipids furnish structural, metabolic, and regulatory roles in several aspects of plant growth, development, and responses to environmental stresses [5,6,7]. Lipids are the major constituents of biological membranes, which act as the interface between the cell and the environment and compartmentalize metabolites into subcellular organelles. The structure and function of cells are dependent on the fluidity and stability of membranes, which are determined by lipid composition and unsaturation levels [8]. A wide range of lipid classes or lipid-derived molecules, such as lysophospholipids, phosphatidic acid (PA), diacylglycerol, triacylglycerol, inositol phosphate, oxylipins, sphingolipids, *N*–acylethanolamine, and sterol lipids have been proposed to function in stress signaling or adaptation mechanisms [9,10,11,12,13,14]. Each of the stress-induced lipid classes plays a specific role in maintaining plant growth and function under stress conditions. Although rarely do biomolecules serve as many diverse roles as lipids, they remain the most poorly characterized molecules in plant biology.

Lipid metabolic changes related to heat stress response have been investigated in plant species such as wheat (*Triticum aestivum* L.) [14,15,16,17,18], maize (*Zea mays* L.) [19], sorghum [*Sorghum bicolor* (L.) Moench] [19], Arabidopsis (*Arabidopsis thaliana*) [20,21,22], and creeping bentgrass (*Agrostis stolonifera*) [23]. A typical response reported to heat stress conditions across species is that the plants decrease lipid unsaturation levels by replacing the highly unsaturated lipids with less unsaturated ones. Authors of earlier studies described these lipid metabolic changes as an adaptation mechanism to heat stress, likely to prevent the phase transition of membranes from a bilayer to non-bilayer phase. However, no conclusive evidence exists in the literature to use any lipid metabolic change as a biomarker for selecting for heat-tolerant genotypes. If the environmentally triggered quantitative and qualitative changes in the lipid profile contribute to heat tolerance, that will have vast implications in crop breeding, particularly for developing molecular markers for heat tolerance.

Fatty acid desaturases (FADs) are enzymes that introduce double bonds (unsaturation) in the fatty acyl chains of lipids. Soybean possesses genes for both extraplastidic and plastidic FADs. The gene that codes for extraplastidic FAD2 exists as a family of eight members, namely *FAD2-1A*, *FAD2-1B*, *FAD2-2A*, *FAD2-2B*, *FAD2-2C*, *FAD2-2D*, *FAD2-2E* and *FAD2-3* [24,25,26,27]. Fatty acid desaturase 2 (oleate desaturase) converts 18:1 extraplastid-localized lipids to 18:2, whereas *FAD6* that exists in a single copy turns 18:1 plastid-localized lipids into 18:2. *Fatty acid desaturase 7* occurs in two copies, namely *FAD7-1* and *FAD7-2* [24,25,26,27], and *FAD8* in a single copy. Both FAD7 and FAD8 convert 18:2 plastid-localized lipids to 18:3. *Fatty acid desaturase 3* occurs in three copies - *FAD3A*, *FAD3B*, and *FAD3C* [24,25,26,27]. Fatty acid desaturase 3 (linoleate desaturase) converts 18:2 extraplastid-localized lipids to 18:3.

## 2. Results

### 2.1. Physiological and Yield Responses of the Soybean Genotypes to Heat Stress

**Figure 1:**
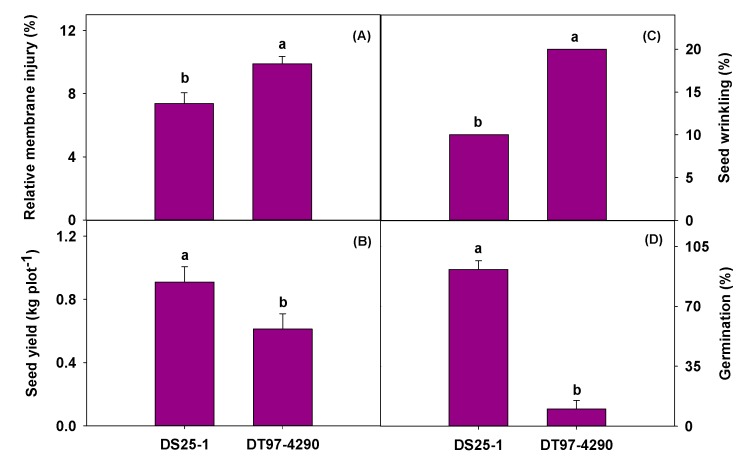
Relative membrane injury in leaves (**A**), seed yield (**B**), seed wrinkling (**C**), and seed germination ability (**D**) of soybean genotypes DS25-1 and DT97-4290 exposed to heat stress. Relative membrane injury, which is a measure of cell membrane stability, was measured on plants grown under controlled environmental conditions. These plants were exposed to heat stress (38/28 °C) for 7 d during the seedling stage. Seed yield, wrinkling, and germination ability were measured on plants grown under field conditions at Stoneville, MS during April–September 2019. Plants experienced > 35/25 °C (supra-optimal temperatures) on >50 days during the life cycle due to hot weather conditions at Stoneville. Seed yield and wrinkling were measured for plants harvested at growth stage, R8 (full maturity), whereas seed germination ability was measured for plants harvested 14 d after growth stage, R8.

Physiological responses of the soybean genotypes to heat stress were quantified based on relative membrane injury (RI), which is an indication of cell membrane stability [28]. Genotype DS25-1 had significantly lower RI than DT97-4290 when grown under heat stress conditions (Figure 1A). This indicates the heat tolerance of DS25-1 and heat susceptibility of DT97-4290 based on a physiological trait (cell membrane stability) that is closely related to membrane lipid composition. Yield responses of the soybean genotypes to heat stress were quantified under field conditions. Seed yield of DT97-4290 was significantly lower than that of DS25-1 when plants experienced heat stress during their life cycle (Figure 1B). Also, seed wrinkling was significantly higher and seed germination ability was significantly lower for DT97-4290, compared to DS25-1 (Figure 1C,D). These results strongly support the heat tolerance of DS25-1 and heat susceptibility of DT97-4290 based on yield and other seed traits measured under field conditions.

### 2.2. Differences in Lipid Metabolic Changes Under Heat Stress Conditions in Soybean Genotypes

Soybean plants were grown under controlled environmental conditions at optimal temperatures (30/20 °C, daytime maximum/night-time minimum) for 15 days. On the 16^th^ day after sowing, two treatments, optimal temperature and heat stress (38/28 °C), were applied for 11 days. Leaf samples were collected for lipid extraction on the 9^th^ day of stress. Direct infusion automated electrospray ionization-tandem mass spectrometry was used to quantitatively profile polar leaf lipids [14].

**Figure 2:**
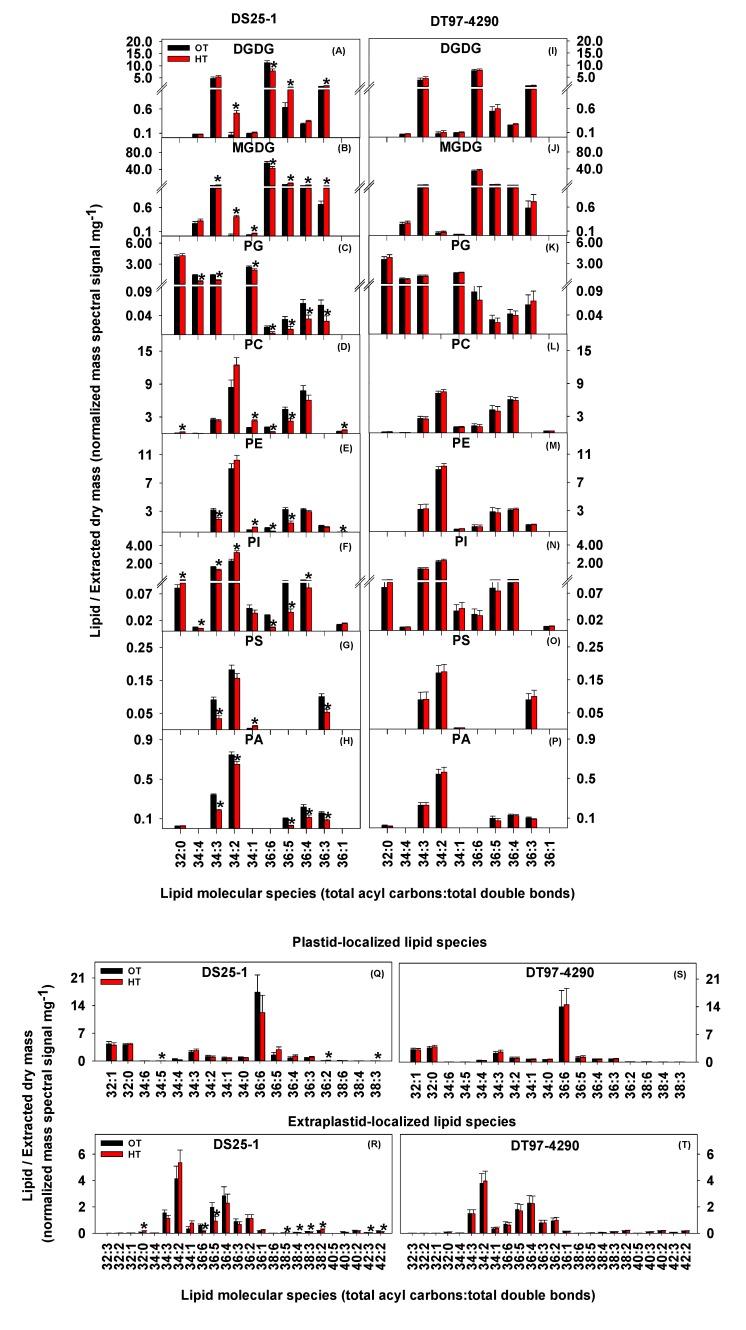
Decrease in the amounts of most unsaturated lipid species (36:6, 36:5, 36:4, 36:3, 34:4, and 34:3 lipids) and increase in the amounts of saturated and less unsaturated lipid species (32:0, 34:1, 34:2, and 36:1 lipids) in the heat-tolerant soybean genotype DS25-1 and absence of these lipid remodeling in the heat-susceptible genotype DT97-4290. Panels **A** through **P** show the effects of temperature on plastid-localized (DGDG, MGDG, and PG) and extraplastid-localized (PC, PE, PI, PS, and PA) diacyl lipid molecular species of DS25-1 (left) and DT97-4290 (right). Panel **Q** through **T** represent summary panels, which show the effects of temperature on plastid-localized and extraplastid-localized lipid species averaged across headgroup classes. Extraplastid-localized lipids are localized in the endoplasmic reticulum. Values shown are least squares means ± SE. Least squares means with ’*’ are significantly different according to the least significant difference (LSD) test at α = 0.05. Breaks on the y-axis indicate a change in scale. OT, optimal temperature (30/20 °C). HT, high temperature (38/28 °C). DGDG, digalactosyldiacylglycerol. MGDG, monogalactosyldiacylglycerol. PG, phosphatidylglycerol. PC, phosphatidylcholine. PE, phosphatidylethanolamine. PI, phosphatidylinositol. PS, phosphatidylserine. PA, phosphatidic acid. The identities of the lipids indicated as total acyl carbons:total double bonds as molecular species with defined acyl chains are: 32:0 (16:0/16:0), 34:4 (18:3/16:1), 34:3 (most likely 16:0/18:3), 34:2 (16:1/18:1 or 16:0/18:2), 34:1 (16:0/18:1), 36:6 (18:3/18:3), 36:5 (18:2/18:3), 36:4 (18:2/18:2 or 18:1/18:3), 36:3 (most likely 18:0/18:3), and 36:1 (18:0/18:1) [29].

**Figure 3:**
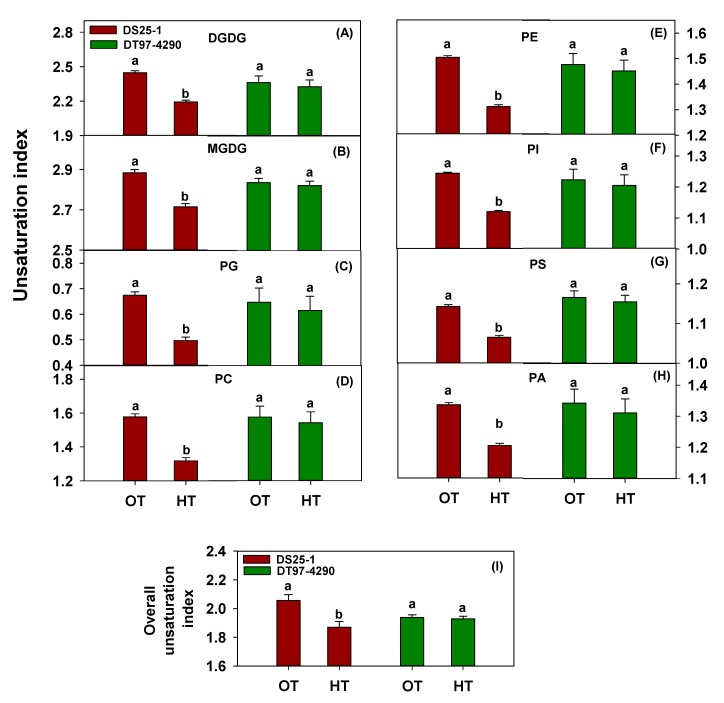
Decrease in unsaturation index under heat stress in the heat-tolerant soybean genotype DS25-1 and no change in unsaturation index under heat stress in the heat-susceptible genotype DT97-4290. Panels **A** through **H** show the effects of temperature on unsaturation index of various lipid head group classes (DGDG, MGDG, PG, PC, PE, PI, PS, and PA). Panel **I** shows the effect of temperature on overall unsaturation index. The unsaturation index of each lipid molecular species was calculated as the average number of double bonds per acyl chain, which is the number of double bonds in the lipid molecular species divided by the number of acyl chains. The unsaturation index of a lipid head group class was calculated as the [sum of (the unsaturation indices of individual lipid molecular species in that class times the amount of each species)] divided by the sum of the amount of lipid molecular species in the class. Values shown are least squares means ± SE. Least squares means with different letters are significantly different according to the least significant difference (LSD) test at α = 0.05. Comparisons have been done between temperature treatments within each genotype. OT, optimal temperature (30/20 °C). HT, high temperature (38/28 °C). DGDG, digalactosyldiacylglycerol. MGDG, monogalactosyldiacylglycerol. PG, phosphatidylglycerol. PC, phosphatidylcholine. PE, phosphatidylethanolamine. PI, phosphatidylinositol. PS, phosphatidylserine. PA, phosphatidic acid.

The heat-tolerant soybean genotype DS25-1 showed decreases in the amounts of some of the most unsaturated lipid species [36:6 digalactosyldiacylglycerol (DGDG), monogalactosyldiacylglycerol (MGDG), phosphatidylglycerol (PG), phosphatidylcholine (PC), phosphatidylethanolamine (PE), and phosphatidylinositol (PI); 36:5 DGDG, MGDG, PG, PC, PE, PI, and PA; 36:4 PG, PI, and PA; 36:3 PG, phosphatidylserine (PS), and PA; 34:4 PG and PI; and 34:3 PG, PE, PI, PS, and PA] and increases in the amounts of saturated or less unsaturated lipid species (32:0 PC and PI; 34:1 MGDG, PC, PE, and PS; 34:2 DGDG, MGDG, PI, and PA; and 36:1 PC and PE) (Figure 2A-H). An overall decrease in 36:6 and 36:5 extraplastid-localized lipids and an overall increase in 32:0 extraplastid-localized lipids were also observed in DS25-1 under heat stress (Figure 2R). On the other hand, no significant changes in the amounts of any individual lipid species were observed in DT97-4290 (Figure 2I-P, 2S,T). Furthermore, DS25-1 showed significantly altered leaf lipid unsaturation levels under heat stress, while the heat-susceptible genotype DT97-4290 exhibited no significant changes. Specifically, under heat stress, DS25-1 showed a decreased unsaturation index for each polar lipid class measured (Figure 3A-H). Consequentially, DS25-1 exhibited an overall decline in the unsaturation index (Figure 3I). At the same time, no measurable change in the unsaturation index of DT97-4290 under heat stress was observed (Figure 3). The overall unsaturation index (Figure 3I) of DS25-1 was lower than that of DT97-4290 under heat stress. Taken together, above results suggest that in DS25-1 the amounts of lipids with 18:3 acyl chains (36:6, 36:5, 36:4, 36:3, 34:4, and 34:3 species) decreased during heat stress, and the amounts of lipids with 18:0, 18:1, 18:2, 16:1, and/or 16:0 acyl chains (32:0, 34:1, 34:2, and 36:1 species) increased. Collectively, these changes led to a decrease in polar lipid unsaturation indices for this genotype.

### 2.3. Expression Level Differences in the Soybean FAD Genes between DS25-1 and DT97-4290 Under Optimal Temperature and Heat Stress Conditions

**Figure 4:**
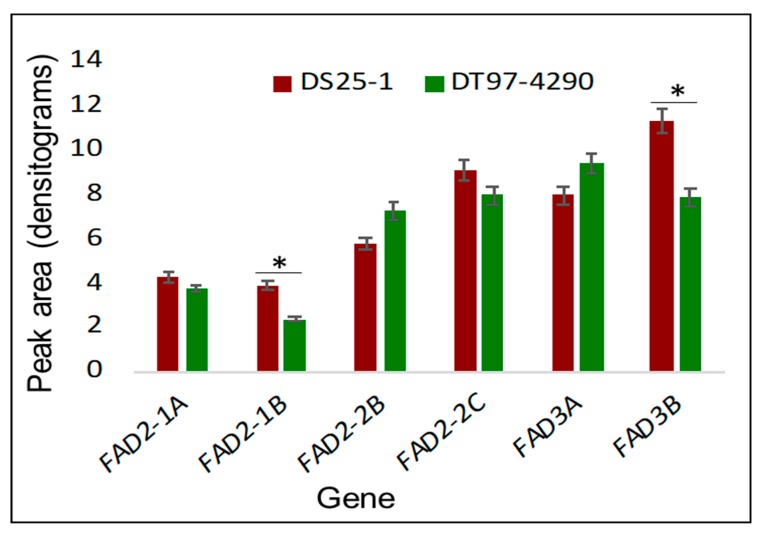
Expression analysis of the *Fatty Acid Desaturase* (*FAD*) genes under optimal temperature conditions in the leaf samples (26 days old) of a heat-tolerant soybean genotype DS25-1 and heat-susceptible soybean genotype DT97-4290. The two soybean genotypes did not show any significant differences in the expression pattern of the *FAD2-1A*, *FAD2-2B*, *FAD2-2C*, and *FAD3A* genes under optimal temperature conditions (30/20 °C). The asterisk signifies a significant difference in the expression level of the *FAD* genes in the two soybean genotypes. Semi-quantitative reverse transcription-polymerase chain reactions (RT-PCRs) were performed using gene-specific primers [24], except for the *FAD3A* and *FAD3B* genes. The *FAD3A* and *FAD3B* genes were amplified together using a primer pair, and the two transcripts were discriminated via restriction digestion of the amplified fragment with the *Van91*I enzyme [25,27] (see Table S1 for primer details and PCR conditions). Following PCR, products were loaded onto agarose gel and the gel images were used for densitometric analysis. In all cases, gene expression results were normalized to the expression of the housekeeping gene. The Student’s *t*-test was used to compare the expression levels of the *FAD* genes in DS25-1 and DT97-4290. The error bars signify the standard error.

Leaf samples for RNA extraction were collected on the 11^th^ day of heat stress. A reverse transcription-polymerase chain reaction (RT-PCR) analysis was conducted to quantify the *FAD* gene expression in soybean leaves. We hypothesized that observed changes in lipid unsaturation levels correspond with expression levels of the *FAD* genes. As mentioned earlier, soybean possesses several omega-6 (*FAD2* and *FAD6*) and omega-3 (*FAD3*, *FAD7*, and *FAD8*) desaturase genes whose products are localized in the plastids or endoplasmic reticulum. There are eight copies of the *FAD2* gene (*FAD2-1A*, *FAD2-1B*, *FAD2-2A*, *FAD2-2B*, *FAD2-2C*, *FAD2-2D*, *FAD2-2E*, and *FAD2-3*), three copies of the *FAD3* gene (*FAD3A*, *FAD3B*, and *FAD3C*), and two copies of the *FAD7* gene (*FAD7-1* and *FAD7-2*) [24,25,26,27]. Other *FAD* genes (*FAD6* and *FAD8*) exist as single copies. Out of these genes, *FAD2-2B*, *FAD2-2C*, *FAD3A*, and *FAD8* are known to exhibit cold-induced expression [25,26,27]. The *FAD2-2A*, *FAD2-2B*, *FAD2-2C*, *FAD2-2D*, *FAD2-3*, *FAD3C*, *FAD7-1*, and *FAD7-2* were reported to be expressed in vegetative tissues, whereas *FAD2-2E* was reported to be expressed exclusively in developing pods [24,25,26,27,30]. Lakhssassi et al. [30] demonstrated via RNA sequencing (RNAseq) experiments that the *FAD2-2D* expresses specifically in the flower, seed, and nodule. Though *FAD2-3* constitutively expresses in both vegetative and developing seed tissues, it was shown to exhibit no changes in transcript abundance in response to growth temperature [31]. Expression of *FAD3C* has been reported in mature leaves [27] and developing seeds [32]. Though a reduction in the level of *FAD3C* expression under heat stress has been reported in developing seeds, the extent of reduction was minimal (close to the detection limit of qRT-PCR (2-3 fold difference, i.e., 1-1.5 PCR cycle)) [32]. Additionally, *FAD3C* did not show a change in expression in response to cold-treatment (5 °C) [27]. Given this prior knowledge, we set out to test the expression of soybean *FAD2-1A*, *FAD2-1B*, *FAD2-2A*, *FAD2-2B*, *FAD2-2C*, *FAD3A*, and *FAD3B* genes in young (26-day old) leaves of soybean genotypes DS25-1 and DT97-4290. We have not tested the expression of any genes for plastidic FADs (e.g., *FAD6*, *FAD7*, and *FAD8*) in this study. The expression of the soybean *FAD2-1A*, *FAD2-1B*, *FAD2-2A*, *FAD2-2B*, and *FAD2-2C* genes was studied using gene-specific primers in RT-PCR (see Materials and Methods). On the other hand, the soybean *FAD3A* and *FAD3B* genes were amplified together using a primer pair, and the two transcripts were recognized via restriction digestion of the amplified fragment with the *Van91*I enzyme [25,27]. All genes except *FAD2-2A* showed expression in the leaves (Figure 4), although genotypic differences in the gene expression levels were observed (Figure 4). It should be noted that this experiment was performed on plants kept in the optimal temperature condition (30/20 °C). In pairwise comparisons, DS25-1 and DT97-4290 did not show significant differences in the expression pattern of the *FAD2-1A*, *FAD2-2B*, *FAD2-2C*, and *FAD3A* genes under optimal temperature conditions (Figure 4).

**Figure 5:**
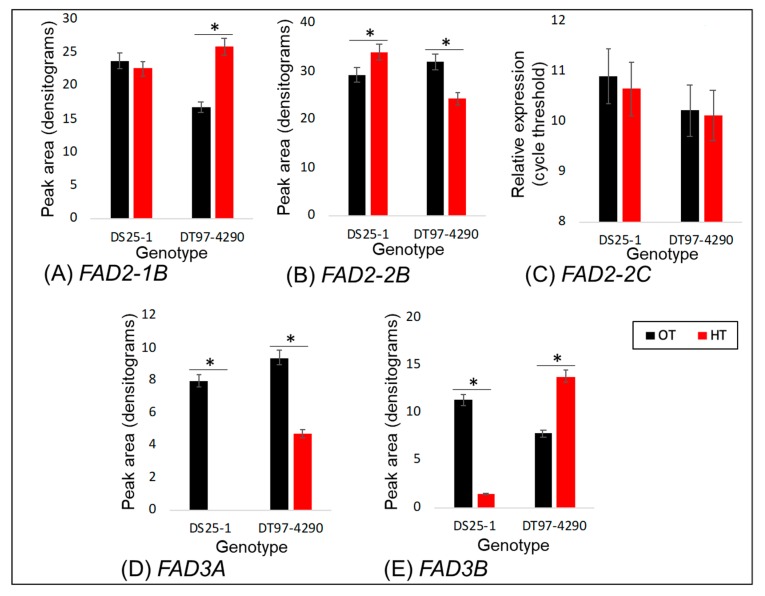
Changes in expression levels of the *Fatty Acid Desaturase* (*FAD*) genes under heat stress in the leaf samples (26 days old) of a heat-tolerant soybean genotype DS25-1 and heat-susceptible soybean genotype DT97-4290. DS25-1 showed significant reductions in expression levels of the *FAD3A* and *FAD3B* genes under heat stress conditions. RT-PCR was performed using gene-specific primers [24,25], and in all cases, gene expression results are normalized to the expression of the housekeeping gene *ACTIN6* (see Supplementary Table S1). For this experiment, plants were grown under optimal temperature (OT; 30/20 °C) or heat stress (HT; 38 °C/28 °C) conditions. For RT-PCR analysis, three biological replicates were used, and for qRT-PCR analysis, three biological and two technical replicates were used. (**A**&**B**) semi-quantitative RT-PCR followed by electrophoresis and densitometric analysis. (**C**) qRT-PCR analysis. (**D**&**E**) semi-quantitative RT-PCR followed by digestion of PCR product with restriction endonuclease *Van*91I, electrophoresis, and densitometric analysis [25,27]. The asterisk signifies a significant difference in the expression level of the *FAD* genes in two soybean genotypes under two treatments. The Student’s *t*-test was used to compare the expression level of the *FAD* genes under two treatments. The error bars signify the standard error.

Under heat stress conditions (38/28 °C), significant changes in the expression levels of the *FAD3A* (expression undetectable, Figure 5D), *FAD3B* (89% decline, Figure 5E), and *FAD2-2B* (16% increase, Figure 5A) genes were observed in the case of DS25-1. However, no significant variation was observed in the expression patterns of the *FAD2-1B* and *FAD2-2C* genes in this genotype (Figure 5B,D). On the other hand, in DT97-4290, the *FAD3A* gene showed a 50% decline in expression level under heat stress, the *FAD3B* gene, a 43% increase, the *FAD2-1B* gene, a 35% increase, and the *FAD2-2B* gene, a 24% decline. As in DS25-1, in DT97-4290, changes in the expression level of the *FAD2-2C* gene with temperature was not significant. The results are consistent with the lipid profiling data in that DT97-4290 did not exhibit the decline in expression of *FAD3B* (whose product forms 18:3), as observed for DS25-1, nor was the level of expression of *FAD3A* (whose product also forms 18:3) lowered in DT97-4290 as much as in DS25-1. Thus, the greater reduction in expression levels of *FAD3*s in DS25-1 as a function of high temperature is consistent with the reduction in the content of 18:3 lipids observed only in DS25-1.

## 3. Discussion

This study examined the changes in unsaturation indices of plastid-localized and extraplastid-localized membrane polar lipids in the leaves of a heat-tolerant soybean genotype DS25-1 and a heat-susceptible soybean genotype DT97-4290 under heat stress conditions. DS25-1 showed a decrease in the unsaturation indices of all measured lipid classes under heat stress conditions, while DT97-4290 did not show any changes. The decline in unsaturation indices in DS25-1 was due to the decreases in the amounts of lipid species that likely had 18:3 acyl chains (36:6, 36:5, 36:4, 36:3, 34:4, and 34:3 DGDG, MGDG, PG, PC, PE, PI, PS, and/or PA). In parallel, increases in the amounts of lipid species with 18:0, 18:1, 18:2, 16:1, and/or 16:0 acyl chains (32:0, 34:1, 34:2, and 36:1 DGDG, MGDG, PG, PC, PE, PI, PS, and/or PA) were observed, which also contributed to an overall decrease in the unsaturation index of DS25-1. Future studies should quantify the changes in 18:3, 18:2, 18:1, 18:0, 16:1, and 16:0 fatty acids under heat stress in DS25-1 to improve the clarity of above observation.

*Cis* double bonds that are commonly present in most plant cell membrane fatty acyl chains introduce bends in the chains and reduce the degree of compact packing of adjacent lipid molecules [33]. The compactness of packing is also reduced by heating. Decreasing the number of double bonds at high temperatures can be adaptive in plants to maintain the optimal lipid packing, fluidity, and integrity of membranes [23]. Our results suggest that decline in the level of lipid unsaturation by decreasing the polyunsaturated fatty acids, such as linolenic acid (18:3), and increasing the less unsaturated fatty acids, such as linoleic (18:2), oleic (18:1), and palmitoleic (16:1) acids, as well as saturated fatty acids such as palmitic (16:0) and stearic (18:0) acids is associated with heat tolerance in DS25-1. Thus, we hypothesized that these changes in lipid profile might correspond to the changes in the expression levels of the *FAD* genes.

As mentioned earlier, soybean possesses a large number of omega-6 (FAD2 and FAD6) and omega-3 (FAD3, FAD7, and FAD8) desaturases that localize to endoplasmic reticulum or chloroplasts. We monitored the expression of the members of two important *FAD*s, one from each class - omega-6 (*FAD2*) and omega-3 (*FAD3*) in the young trifoliate leaves of the soybean genotypes. The expression level differences of *FAD2-1A*, *FAD2-1B*, *FAD2-2B*, *FAD2-2C*, and *FAD3A* genes in both genotypes under optimal temperature conditions were subtle (Figure 4). This is not unexpected, given that FAD function is required in all plant tissues and throughout development to produce unsaturated fatty acids required for normal membrane and photosynthetic functions. No expression of the *FAD2-2A* gene was recorded in the leaves, which corroborate with the previous findings [24].

Under heat stress, DS25-1 and DT97-4290 exhibited differential expression patterns for the *FAD3* genes (Figure 5D,E). DS25-1 showed a stark reduction in the expression level of both *FAD3A* and *FAD3B* genes in the leaves. These results are consistent with the lipid profiling data, where a reduction in the content of 18:3 lipids was observed in DS25-1. Since the *FAD3* genes encode linoleate desaturase, which converts 18:2 to 18:3 lipids, a decline in the *FAD3* expression level is expected to reduce the content of polyunsaturated (18:3) lipids. More evidence in support of this hypothesis could be gathered by monitoring differences in accumulation of encoded proteins and their lipid products and/or studying the effects of these changes on membrane composition and functioning. Post-translational regulation of the FADs and the transcriptional regulation by a feedback or other mechanism cannot be denied at this time point. These possibilities will be tested in future studies. The present research suggests a role of the *FAD3* genes in differential responses of DS25-1 and DT97-4290 to heat stress. This introduces the possibility of testing reduced *FAD3* activity as a biomarker for heat tolerance in soybean. Future studies need to evaluate soybean populations under heat stress conditions and assess whether the expression level differences of *FAD3* translate to the protein level and further to lipid accumulation.

Meta-expression analysis of the *FAD2-1*, *FAD2-2*, *FAD2-2-like*, *FAD4*, *FAD5*, *FAD6*, and *FAD8* genes performed on the existing soybean microarray and RNAseq data using Genevestigator version 7.2.6 [34] suggested that the *FAD2-2* genes express throughout plant development with a peak during early development (primarily leaves) (Figure S1A,B). In contrast, *FAD2-1* was expressed predominantly later during plant development, and specifically in pods and seeds (Figure S1C,D). These observations corroborate the present findings, where the *FAD2-2B* and *FAD2-2C* genes showed higher expression level than the *FAD2-1A* and *FAD2-1B* genes in the leaf tissue collected from 26-day-old plants (Figure 4).

The present study has important implications in soybean breeding. If reduced *FAD3* activity and the resulting decrease in unsaturation levels under high temperature conditions are associated with heat tolerance in soybean, *FAD3* can serve as a molecular marker for selecting for heat-tolerant genotypes. Currently, the major limitation for soybean breeding programs in developing heat-tolerant varieties is the lack of high throughput selection criteria for tolerant genotypes. To the knowledge of the authors, to date, no heat-tolerant soybean variety has been developed and released by introducing a specific tolerance gene or by using a molecular marker-assisted selection. The major reason is the polygenic nature of heat tolerance, which makes it difficult to identify a single gene conferring that trait. This shows the importance of identifying a gene related with lipid metabolism, which is strongly correlated with heat tolerance in soybean.

The present study reports results on two soybean genotypes with contrasting levels of heat-tolerance. However, this analysis was performed at a single developmental stage after exposing the seedlings to heat stress. It corroborates with the earlier finding, where the correspondence between heat-induced suppression of the *FAD3* expression level and seed linolenic acid content was observed [32]. In that study, Byfield and Upchurch [32] used three soybean varieties (‘Dare,’ ‘N01-3544′, and ‘N99-3170′) with different seed linoleic and linolenic contents and exposed them to heat stress during seed development. The current study provides convincing evidence that heat-induced suppression of the *FAD3* expression corresponds to low linolenic acid content, which in turn contributes to heat tolerance in DS25-1. The utility of the *FAD3* expression level as a marker will be validated further in a future study using a bi-parental mapping population derived from a cross between DS25-1 and DT97-4290, and soybean *FAD3A* and *FAD3B* mutant lines ‘CX1512-44′ [35] and ‘A29′ [36], respectively. These future studies will correlate the heat tolerance phenotype with the changes in gene expression and lipid profile through time. However, the current results serve as the rationale for the long-term objective of identifying lipid-related molecular markers for heat tolerance in soybean.

## 4. Materials and Methods

### 4.1. Plant Material

Soybean genotype DS25-1 (breeding line designation, 04025-1-1-4-1-1) was developed from a cross between DT98-9102 and PI 587982A, and released by the USDA in 2017 [37]. Genotype PI 587982A was donated to the U.S. National Plant Germplasm System in 1994 by the Chinese Academy of Agricultural Sciences and was identified as having consistent and robust heat tolerance (maintenance of seed weight and high germinability in high-temperature environments) [38]. Transcriptomic analysis found differentially expressed genes under heat stress for PI 587982A compared with a conventional high-yielding line [39]. Genotype DT97-4290 was developed from a cross between Asgrow A5979 and Delta Pine DP3478. It was originally released for its resistance to charcoal rot, southern stem canker, Soybean Mosaic Virus, and Races 2, 4, and 10 of Phytophthora rot [40]. The effects of temperature on seed quality of genotypes DS25-1 and DT97-4290 were tested under controlled environmental conditions [41]. When moderate (36/24 °C, daytime maximum/night-time minimum) or severe (42/26 °C) heat stress conditions were imposed on the plants from flowering to maturity, the harvested seeds of DS25-1 had greater germination percentage and radicle length than the seeds of DT97-4290 [41].

The current authors conducted additional experiments to quantify physiological and yield responses of the soybean genotypes to heat stress. In order to determine the differences in cell membrane stability between DS25-1 and DT97-4290 under heat stress, five plants (five replications) of each genotype were grown under controlled environmental conditions at Clemson University, Clemson, South Carolina, USA in 2019. Plants were maintained under optimal temperature conditions (30/20 °C) for 12 d. On the 13^th^ day after sowing, plants were moved to a growth chamber (Caron model 7300-75-2, Marietta, OH, USA) set at 38/28 °C (heat stress).

Leaf cell membrane stability was measured on the 7^th^ day of the heat treatment period using the protocol given by Martineau et al. [28]. Briefly, leaf discs of 6 mm diameter were collected from the youngest, fully expanded leaf from each plant using a hole puncher. Four piles of six leaf discs were made from each plant. Each pile was placed in a test tube containing 10 mL of deionized water. Leaf discs in each test tube were washed with four changes of deionized water to remove electrolytes released from cut cells at the periphery of the discs and any exogenous electrolytes on the leaves. After the final wash, excess water was drained, leaving the wet leaf discs at the bottom for each test tube. At that point, all test tubes were covered with plastic wrap and aluminum foil. Two of the four test tubes for each plant were placed in a water bath at 55 °C (treatment, T) and the other two were placed in another water bath at 25 °C (control, C) for 15 minutes. Afterwards, 30 ml of deionized water was added to each test tube and all test tubes were moved to a 10 °C incubator and allowed to stay there for 18 hours to let the diffusion of electrolytes from the discs. Then, the test tubes were moved to a 25 °C incubator to bring them back to room temperature. Once at room temperature, each test tube’s content was mixed before taking the initial electrical conductivity reading with a conductivity meter (VWR^®^ Traceable^®^ Expanded Range Conductivity Meter, Radnor, PA, USA). The electrical conductivity readings for the two test tubes per plant placed at 55 °C or 25 °C were averaged to get a single value for T and C. After taking the initial conductivity reading for all test tubes, the test tubes were covered with aluminum foil and autoclaved at 120 °C for 15 minutes to kill the leaf tissue and release all electrolytes from the leaf discs. After cooling down to room temperature, a final electrical conductivity reading was taken for each test tube. Relative injury (RI) for each plant was calculated as Equation (1):

RI (%) = [1-(1-T_1_⁄T_2_)/(1-C_1_⁄C_2_)]×100, (1)

where the subscripts 1 and 2 represent the initial and final electrical conductivity readings, respectively. Relative injury is related to cell membrane stability such that the higher the RI, the lower the cell membrane stability [28]. GLIMMIX procedure in SAS (Version 9.4, SAS Institute) was used to perform analysis of variance and to estimate least squares means and standard errors. Separation of least squares means was done based on the LSD test using the LSMEANS option in the GLIMMIX procedure.

To quantify the heat responses of the genotypes, DS25-1 and DT97-4290, based on yield and other seed traits, they were sown under field conditions at Stoneville, MS on 22 April 2019. Both genotypes were grown in single row (2.7 m length) plots, which were arranged in a randomized complete block design with two replications (each block included 213 other genotypes than DS25-1 and DT97-4290, but here we are presenting data only for these two genotypes). Row spacing was 91 cm and plant-to-plant spacing within the rows was 3.8 cm. All plots were maintained under rain-fed conditions. Both genotypes experienced > 35/25 °C (supra-optimal temperatures) on >50 days during the life cycle (Figure S2). Especially, the hot weather during late August and September led to significant heat stress during late pod fill and senescence for both genotypes (Figure S2). Plants were hand-harvested at growth stage R8 (full maturity) and then threshed using a small-plot bundle thresher. Genotype DT97-4290 was harvested on 09/10/2019, whereas, DS25-1 was harvested on 9/18/2019. Seeds were rated for wrinkling as described by Smith et al. [38] and Kebede et al. [42]. Briefly, wrinkled seed ratings were taken on the bulked seed of each plot as the percentage of visibly wrinkled seed coat surface area per total visible seed coat surface area. Seed coat wrinkling is a major factor affecting the germinability of soybean seed produced under heat stress conditions [42].

Data were also collected on germination ability of DS25-1 and DT97-4290 exposed to heat stress under field conditions. For this purpose, plants of DS25-1 and DT97-4290 were grown at Stoneville, MS with the same protocol as described above, but in a separate experiment. Plants were harvested 14 d after growth stage R8 in order to let plants weather under the hot field conditions. Assays for standard germination were carried out by the Mississippi Bureau of Plant Industry State Seed Testing Laboratory following the protocols of the Association of Official Seed Analysts [43]. A 200-seed sample was randomly taken from each plot following harvest and threshing and sent to the seed testing lab. For each plot, a mean of four samples of 50 seeds each was used as the estimate for standard seed germination.

GLIMMIX procedure in SAS (Version 9.4, SAS Institute) was used to perform analysis of variance for the field experiments and to estimate least squares means and standard errors. Separation of least squares means was done based on the LSD test using the LSMEANS option in the GLIMMIX procedure.

### 4.2. Plant Husbandry and Stress Treatment for the Lipid and Gene-Expression Experiments

Soybean plants were grown under controlled environmental conditions at Clemson University, Clemson, South Carolina, USA in 2019. Seeds of the genotypes DS25-1 and DT97-4290 were inoculated with the fungicide BeanGuard^®^/Allegiance^®^ [captan (N-[(trichloromethyl)thio]-4-cyclohexene-1,2-dicarboximide)-carboxin (5,6-dihydro-2-methyl-N-phenyl-1,4-oxathiin-3-carboxamide)-metalaxyl (N-(2,6-dimethylphenyl)-N-(methoxyacetyl)alanine methyl ester] before sowing at the rate of 2 g kg^-1^. Seeds were sown in 7.6-L pots containing potting soil (Fafard^®^3B Mix/Metro-Mix^®^830, SUNGRO Horticulture, Agawam, MA, USA) in a greenhouse on 15 February 2019. The potting soil was fertilized with Osmocote, a controlled-release fertilizer with 18:6:12, N:P_2_O_5_:K_2_O at 25 g per pot before sowing. A systemic insecticide, Marathon (a.i.: Imidacloprid: 1-[(6-Chloro-3-pyridinyl)methyl]-N-nitro-2-imidazolidinimine; OHP, Inc., Mainland, PA, USA) was also applied to the potting soil before sowing at 4.5 g per pot to avoid infestation of sucking insect pests. There were 10 plants of each genotype in the greenhouse, which were maintained under optimal temperature conditions (30/20 °C) for 15 d. Photosynthetically active radiation (400 to 700 nm) at the top of the plant canopy was ~ 675 μmol m^-2^ s^-1^ in the greenhouse. The average relative humidity was set at 75% in the greenhouse, and the observed values were 30%/41% (average day/night). On the 16^th^ day after sowing, two treatments, optimal temperature and heat stress (38/28 °C), were applied for 11 days. To impose heat stress, half of the plants of both genotypes were moved to a growth chamber (Caron model 7300-75-2, Marietta, OH, USA) set to 38/28 °C (daytime maximum/night-time minimum). The other half of the plants remained in the greenhouse and received optimal temperatures. The maximum and minimum temperatures were held for 12 h in the growth chamber. Photosynthetically active radiation (400 to 700 nm) provided by cool fluorescent lamps at the top of the plant canopy was 600 μmol m^-2^ s^-1^ in the growth chamber, and the photoperiod was 12 h. The average relative humidity was set at 75% in the growth chamber, and the observed values were 69%/87% (average day/night). After the stress period, plants were returned to the greenhouse (30/20 °C), where they remained until the final harvest at physiological maturity.

In both greenhouse and growth chamber, air temperature was continuously monitored at 15 min intervals using a HOBO data logger (Onset Computer Corporation, Bourne, MA, USA). The quality of temperature control is shown in Figure S3. Throughout the experiment, plants were maintained as well-watered and the position of pots was changed randomly in 1-5 d interval to avoid positional effects.

### 4.3. Lipid Extraction

For lipid extraction, leaf samples were collected from five plants per genotype from optimal and high temperature conditions between 09:30 and 10:30 hours on the 9^th^ day of stress or at the same time point for plants at optimal temperature conditions. At sampling, the middle leaflet from the top trifoliate (fully opened top-most trifoliate that has a visible node) was cut and immediately chopped into 6 mL of isopropanol with 0.01% butylated hydroxytoluene (BHT) at 75 °C in a 50 mL glass tube with a Teflon lined screw-cap (DWK Life Sciences L.L.C., Millville, NJ, USA). Tubes were kept at 75 °C for 15 min to deactivate lipid-hydrolyzing enzymes. After cooling the samples to room temperature, 3 mL of chloroform and 1.2 mL of water were added, and samples were stored at –80 °C until analysis. The lipid extraction procedure was carried out as previously described [14]. Briefly, the lipid extract in isopropanol, BHT, chloroform, and water was shaken on an orbital shaker at room temperature for 1 h and transferred to a new glass tube using a Pasteur pipette, leaving the leaf pieces at the bottom of the original tube. Four mL of chloroform:methanol (2:1) with 0.01% BHT were added to the leaves, the samples were shaken on an orbital shaker at room temperature overnight and the solvent was transferred to the first extract. The addition, shaking (overnight), and transfer steps were performed four times until the leaf pieces of every sample appeared white. At this stage, the solvent was evaporated from the extract in an N-EVAP 112 nitrogen evaporator (Organomation Associates, Inc., Berlin, MA, USA). The lipid extract was dissolved in 1 mL of chloroform, transferred to a 2 mL clear glass vial with a Teflon-lined screw cap (DWK Life Sciences L.L.C., Millville, NJ, USA), and stored at -80 °C. In order for shipping, the solvent (chloroform) was again evaporated from the lipid extract in the N-EVAP 112 nitrogen evaporator. The vials containing the lipid extracts were shipped overnight to Kansas Lipidomics Research Center (KLRC) with dry ice. At KLRC, the lipid extract was again dissolved in 1 mL of chloroform and was used for lipid profiling (see below). The extracted leaf pieces were dried in an oven at 105 °C overnight, cooled and weighed to express the lipid content on a dry weight basis. Dry weights were determined using a balance (Mettler Toledo ME104E, Mettler Toledo International, Inc., Columbus, OH, USA), which had a detection limit of 0.1 mg. All samples exceeded a weight of 1 mg.

### 4.4. Electrospray Ionization-Triple Quadrupole Mass Spectrometry Lipid Profiling

An automated electrospray ionization-tandem mass spectrometry approach was used, and data acquisition and analysis and acyl group identification were carried out as previously described [14]. The profiled lipid molecular species belonged to digalactosyldiacylglycerol (DGDG), monogalactosyldiacylglycerol (MGDG), phosphatidylglycerol (PG), phosphatidylcholine (PC), phosphatidylethanolamine (PE), phosphatidylinositol (PI), phosphatidylserine (PS), PA, lysophosphatidylglycerol (LPG), lysophosphatidylethanolamine (LPE), and lysophosphatidylcholine (LPC) head group classes. The lipid molecular species were identified by precursor or neutral loss scanning, and the lipids in each head group class were quantified in comparison with internal standards of that class (given in Table S1 in Narayanan et al. [14]). The goal of the quantification was to compare different leaf samples for the amount of each lipid molecular species, rather than to compare the absolute amounts of various lipid molecular species with each other. To assure that the data for each molecular species could be compared throughout the mass spectral data acquisition periods, a quality-controlled approach was employed [14,44,45]. Quality control (QC) samples were prepared by pooling an aliquot from each lipid sample and were analyzed recurrently among the experimental samples. The intensity of each lipid species in the experimental samples was normalized using the QC analyte intensities, as previously described [14]. The lipid values were reported as normalized intensity per mg leaf dry weight, where a value of one is the intensity of 1 nmol of internal standard (See Dataset S1). To maintain data quality, the following data were removed from the data set; (a) lipid analytes for which the amount (normalized mass spectral signal) per milligrams of leaf dry weight was less than the limit of detection and (b) lipid analytes with coefficient of variation (standard deviation divided by mean of the amount of the analyte in the QC samples) greater than 0.3.

### 4.5. Estimation of Lipid Unsaturation Index

Unsaturation index refers to the number of double bonds in a lipid, such that the greater the unsaturation index, the greater is the number of double bonds (degree of unsaturation) in that lipid. The unsaturation index of each lipid molecular species was calculated as the average number of double bonds per acyl chain, which is the number of double bonds in the lipid molecular species divided by the number of acyl chains. The unsaturation index of a lipid head group class was calculated as the [sum of (the unsaturation indices of individual lipid molecular species in that class times the amount of each species)] divided by the sum of the amount of lipid molecular species in the class [14,17,46].

### 4.6. Statistical Analysis

The experiment was conducted with a completely randomized design with a split-plot treatment structure. Temperature was the main plot factor (two levels, optimal temperature and heat stress) and genotype was the split plot factor (genotypes DS25-1 and DT97-4290). There were five replications (five plants, biological replications) for the split-plot treatment factor, genotype. GLIMMIX procedure in SAS (Version 9.4, SAS Institute) was used to perform analysis of variance and to estimate least squares means and standard errors. Separation of least squares means was done based on the LSD test using the LSMEANS option in the GLIMMIX procedure.

### 4.7. RNA Isolation, cDNA Synthesis, and RT-PCR Analysis

Two leaflets flanking the central leaflet of the top trifoliate were collected for RNA extraction on the 11^th^ day of heat stress treatment (38/28 °C) from 26-day-old plants. Total RNA was extracted and purified from 150 mg of flash-frozen tissue using the Spectrum^TM^ Plant Total RNA Kit (Sigma-Aldrich Co. LLC, St. Louis, MO) following the manufacturer’s instructions, except that the RNA was treated with TURBO^TM^ DNase (Life Technologies Corporation, Carlsbad, CA) both on and off the column to eliminate genomic DNA contamination. Following extraction, RNA was quantified using a ND-1000 spectrophotometer (NanoDrop, Wilmington, DE) and converted to cDNA using RevertAid First Strand cDNA Synthesis Kit, (Thermo Fisher Scientific Inc., Waltham, MA). For cDNAs synthesis, 1.5 µg RNA was used following the manufacturer’s instructions.

The expression patterns of the *FAD2-1A*, *FAD2-1B*, *FAD2-2A*, *FAD2-2B*, *FAD3A,* and *FAD3B* genes were examined by a semi-quantitative RT-PCR assay followed by densitometric analysis. Since the gene-specific primers for the *FAD3* gene amplify both *FAD3A* and *FAD3B* genes, restriction digestion was performed with the *Van91*I enzyme (NEB) of the PCR product to distinguish *FAD3A* and *FAD3B* genes [27]. The amplified products for all genes were resolved by electrophoresis on 1% agarose gels. Semi-quantification of the relative gene expression levels was performed through normalization against the housekeeping gene (*ACTIN6*). Densitometric quantification of the PCR bands under non-saturating conditions was performed using ImageJ software [47]. Specific primers used to amplify the *FAD* genes and the *ACTIN6* gene and their respective annealing temperatures, are summarized in Table S1. The amplification reaction was carried out using Ex-*Taq* DNA Polymerase (TaKaRa) according to the manufacturer’s instructions.

The expression patterns of the *FAD2-2C* gene was studied by the real-time quantitative PCR (qPCR) analysis using the iTaq Univer SYBR Green Supermix chemistry on iCycler iQ™ from BioRAD as previously described [48]. PCR primers for the *FAD2-2C* gene and the *ACTIN6* gene (used as internal controls) are listed in Supplementary Table S1. In this case, *FAD2-2C* mRNA level was normalized to *ACTIN6* using the Delta-Delta-CT (DDCT) method [49,50]. Finally, the transcript levels were expressed as a ratio of *FAD2-2C* transcripts (normalized to *ACTIN6*) in DS25-1 and DT97-4290.

## 5. Conclusions

The present study evaluated the changes in leaf polar lipid unsaturation levels in a heat-tolerant soybean genotype DS25-1 and a heat-susceptible soybean genotype DT97-4290 under heat stress and assessed the related changes in the expression levels of the *FAD* genes in order to determine whether changes in lipid unsaturation levels are associated with heat tolerance of DS25-1. We found that decrease in the level of lipid unsaturation due to decrease in the polyunsaturated fatty acid, linolenic acid (18:3) is associated with heat tolerance of DS25-1. In parallel, increases in the amounts of less unsaturated fatty acids, linoleic (18:2), oleic (18:1), and palmitoleic (16:1) acids and saturated fatty acids, palmitic (16:0) and stearic (18:0) acids were observed, which also contributed to an overall decrease in the unsaturation index in DS25-1. Along with the decline in the amount of linolenic acid under heat stress conditions in DS25-1, there was also a reduction in the expression levels of the *FAD3* genes that convert 18:2 extraplastid-localized lipids to 18:3. It suggests that reduced FAD activity could be a potential biomarker for heat tolerance in soybean. Further studies are required to verify the utility of *FAD3* for marker-assisted selection in soybean breeding programs to develop heat-tolerant varieties.

## Data Availability

The lipid data generated or analyzed during this study are included in this published article and its Supplementary Dataset 1. The gene expression data generated and/or analyzed in the current study will be made available to the requestor on the reasonable request.

## Supplementary Materials

The following are available online at https://www.mdpi.com/2223-7747/9/4/457/s1, Dataset S1: Data on 100 lipid species as normalized intensity per mg of leaf dry weight, Table S1: List of the gene-specific primers used for the semi-quantitative and qRT-PCR analyses, Figure S1: Meta-expression analysis of the *Fatty Acid Desaturase* (*FAD*) genes performed on the existing soybean microarray and RNAseq data using Genevestigator, Figure S2: Daily maximum and minimum air temperatures during the soybean growing period in Stoneville, MS, Figure S3: The observed air temperature inside the greenhouse and growth chamber.
