# Supplementary material for: Comparative Lipidomic Analysis Reveals Heat Stress Responses of Two Soybean Genotypes Differing in Temperature Sensitivity
Source: Plants (Basel). 2020 Apr 4;9(4):457. doi: 10.3390/plants9040457 (PMC7238245; doi:10.3390/plants9040457)
Supplement: Supplementary file 1 [file plants-09-00457-s001.zip › plants-754106-proof back/Supplementary Information_Narayanan et al.docx]

**Supplementary Materials**

**Table S1. List of the gene-specific primers used for the semi- quantitative and qRT-PCR analyses.**

| **Primer name** | **Sequence** | **Product size (bp)** | **Annealing temperature (°C)** | **Reference** | |
| --- | --- | --- | --- | --- | --- |
| Semi-quantitative RT-PCR and densitometric analysis | | | | | |
| FAD2-1A_F | GATTTCCTGAAGGCTTAGGTG | 452 | 50 | Schlueter et al. 2007 | |
| FAD2-1A_R | GAAAGCAGTGTGGTGGAATTG |  |  |  | |
| FAD2-1B_F | GATTTCTTGAAGGCTTATGGTAT | 480 | 46 | Schlueter et al. 2007 | |
| FAD2-1B_R | AAGGTCATAAACAACATAGGAC |  |  |  |  |
| FAD2-2A_F | CTTCCACAACCTTCCTCATC | 335 | 42 | Schlueter et al. 2007 | |
| FAD2-2A_R | GGATTGTTTAGGTATTTAGATAG |  |  |  |  |
| FAD2-2B_F | AGTATGCTATGGCCTTTTCTG | 494 | 49 | Schlueter et al. 2007 | |
| FAD2-2B_R | ACCATCCCACCTGACGGAC |  |  |  |  |
| ACTIN-F* | ATTGTAGGTCGTCCTCGTC | 334 | 46 | Román et al. 2012 | |
| ACTIN-R* | TTGCATAAAGTGAAAGAACAG |  |  |  |  |
| Realtime quantitative RT-PCR | | | | |  |
| FAD2-2C_F | ACCTCACCATAGCCTTCTAC | 336 | 44 | Schlueter et al. 2007 | |
| FAD2-2C_R | TATTTAGAGTACCACTTGATACA |  |  |  | |
| Semi-quantitative RT-PCR, restriction digestion, and densitometric analysis | | | | |  |
| FAD3A+B_F | GCAATGGTTAAAGACACAAAGCCT | 1142 | 53 | Román et al. 2012 | |
| FAD3A+B_R | ACTCAGTCTCGGTGCGAGTG |  |  |  |  |

**Actin* was used as control in all experiments

Schlueter, J.A.; Vasylenko-Sanders, I.F.; Deshpande, S.; Yi, J.; Siegfried, M.; Roe, B.A.; Schlueter, S.D.; Scheffler, B.E.; Shoemaker, R.C. The FAD2 gene family of soybean: insights into the structural and functional divergence of a paleopolyploid genome. Plant Genome 2007, 47, S-14-S-26, doi:10.2135/cropsci2006.06.0382tpg.

Román, Á.; Andreu, V.; Hernández, M.L.; Lagunas, B.; Picorel, R.; Martínez-Rivas, J.M.; Alfonso, M. Contribution of the different omega-3 fatty acid desaturase genes to the cold response in soybean. J. Exp. Bot. 2012, 63, 4973-4982, doi:10.1093/jxb/ers174.


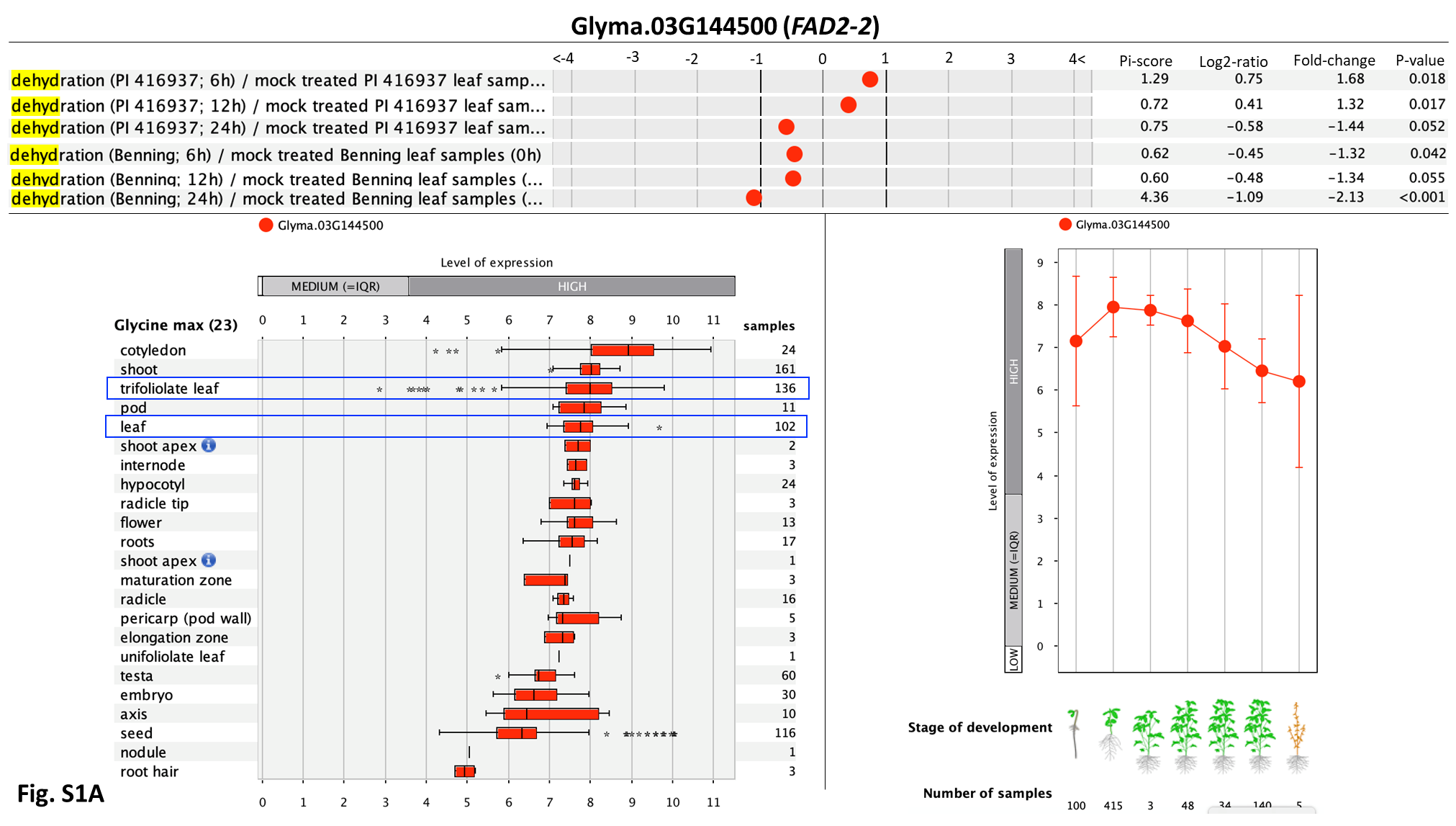


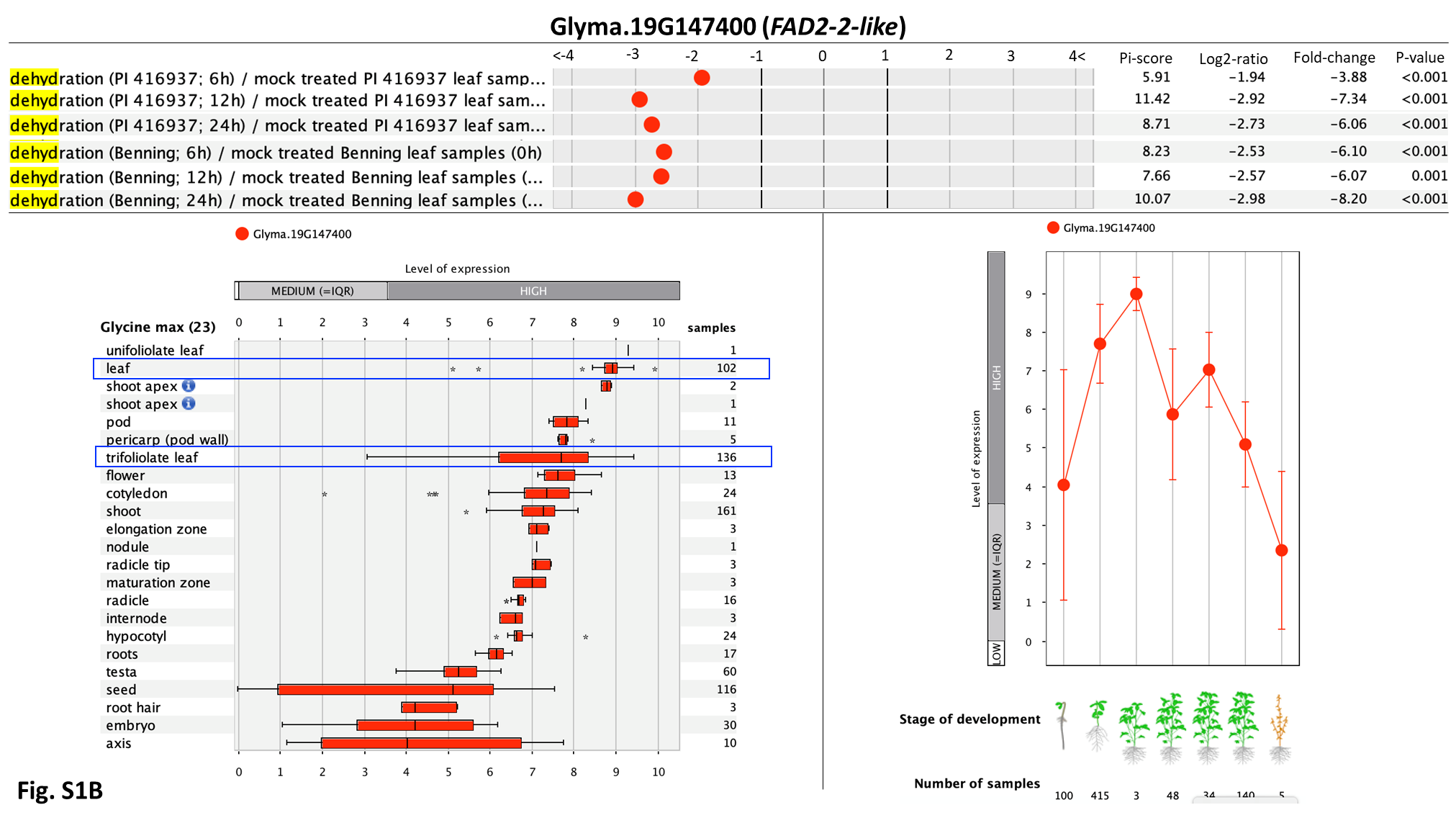


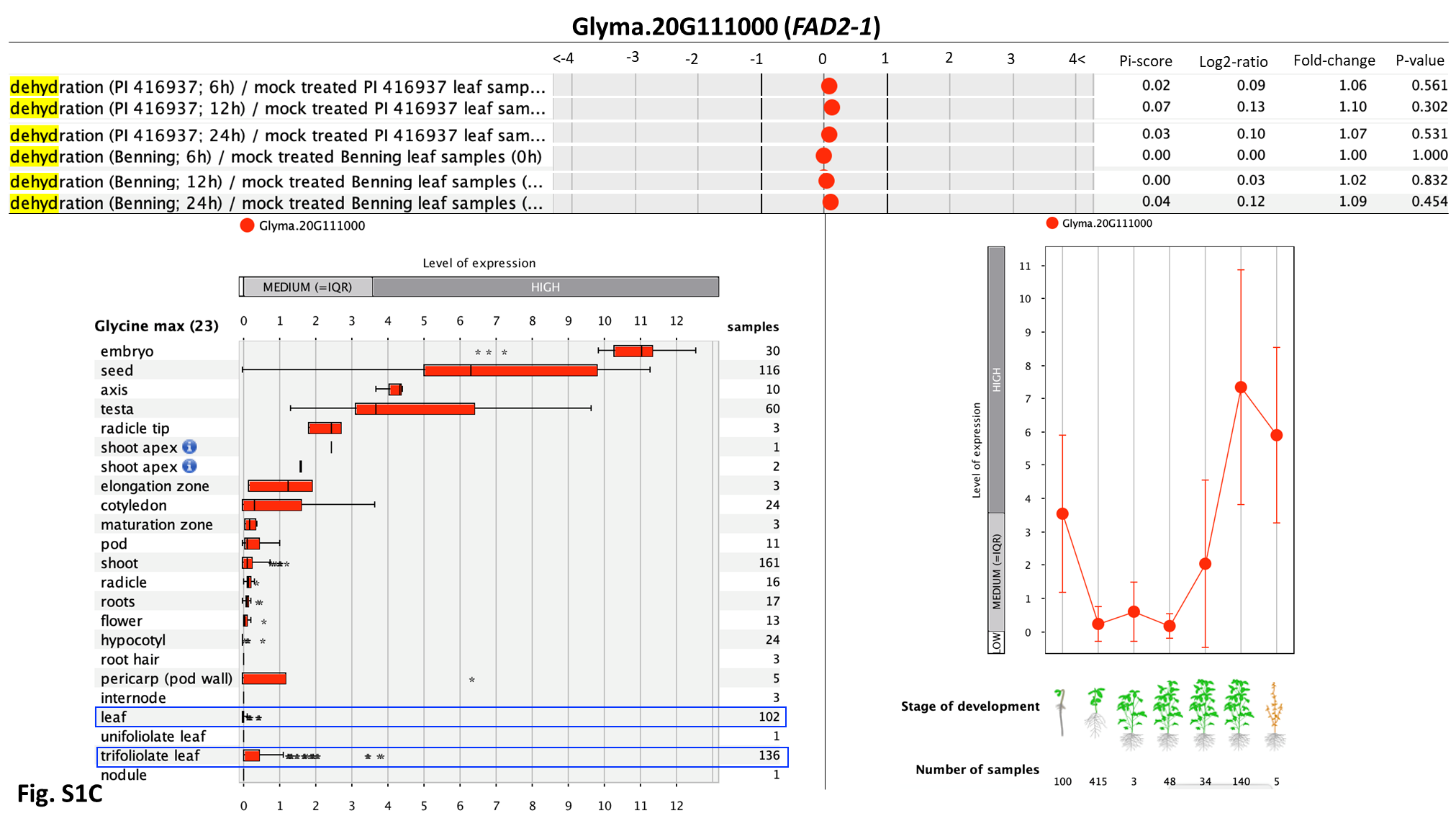


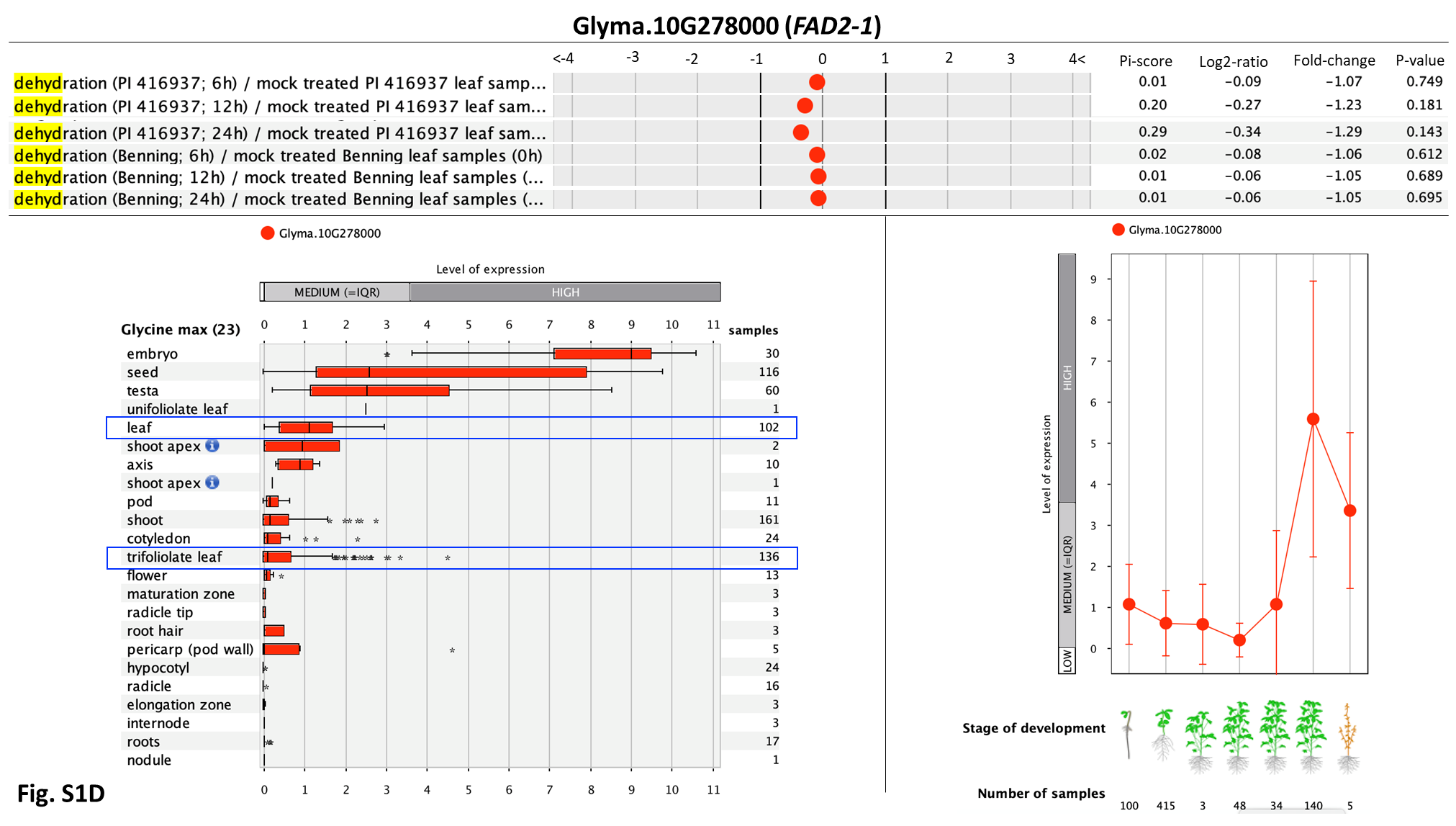


**Figure S1. Meta-expression analysis of the *Fatty Acid Desaturase* (*FAD*) genes performed on the existing soybean microarray and RNAseq data using Genevestigator version 7.2.6. (A)** Glyma.03G144500 (*FAD2-2*), **(B)** Glyma.19G147400 (*FAD2-2-like*), **(C)** Glyma.20G111000 (*FAD2-1*), **(D)** Glyma.10G278000 (*FAD2-1*).

**Figure S2.** **Daily maximum and minimum air temperatures during the soybean growing period in Stoneville, MS.** The weather data were obtained from the Mississippi State Extension Weather website. Soybean genotypes DS25-1 and DT97-4290 were sown on 22 April 2019. Genotype DS25-1 reached flowering stage (R1) on 06/13/2019, full seed stage (R6) on 07/29/2019, and full maturity stage (R8) on 9/16/2019. Genotype DT97-4290 reached flowering stage (R1) on 05/28/2019, full seed stage (R6) on 07/21/2019, and full maturity stage (R8) on 9/8/2019. Genotype DS25-1 was harvested on 9/18/2019, whereas, DT97-4290 was harvested on 09/10/2019.

**Figure S3.** **The observed air temperature inside the greenhouse (A) and growth chamber (B).** The daytime maximum/night-time minimum temperatures were set to 30/20°C (optimal temperature) in the greenhouse and 38/28°C (heat stress) in the growth chamber. High daytime temperature was provided for ~ 12 h from 07:00 to 19:00 hours in the growth chamber. Similarly, high night-time temperature was provided for ~ 12 h from 00:00 to 07:00 hours and 19:00 to 23:59 hours.
